# AflSte20 Regulates Morphogenesis, Stress Response, and Aflatoxin Biosynthesis of *Aspergillus flavus*

**DOI:** 10.3390/toxins11120730

**Published:** 2019-12-13

**Authors:** Ding Li, Ling Qin, Yinchun Wang, Qingchen Xie, Na Li, Shihua Wang, Jun Yuan

**Affiliations:** Key Laboratory of Pathogenic Fungi and Mycotoxins of Fujian Province, Key Laboratory of Biopesticide and Chemical Biology of Education Ministry, and School of Life Sciences, Fujian Agriculture and Forestry University, Fuzhou 350002, China; echoliding@fafu.edu.cn (D.L.); lingqin@fafu.edu.cn (L.Q.); Annewangyinchun@fafu.edu.cn (Y.W.); qingchenxie9@m.fafu.edu.cn (Q.X.); lina3180515023@fafu.edu.cn (N.L.)

**Keywords:** aflatoxin, *Aspergillus flavus*, HOG pathway, *Aflste20*

## Abstract

Various signaling pathways in filamentous fungi help cells receive and respond to environmental information. Previous studies have shown that the mitogen-activated protein kinase (MAPK) pathway is phosphorylation-dependent and activated by different kinase proteins. Serine/threonine kinase plays a very important role in the MAPK pathway. In this study, we selected the serine/threonine kinase AflSte20 in *Aspergillus flavus* for functional study. By constructing *Aflste20* knockout mutants and complemented strains, it was proven that the *Aflste20* knockout mutant (Δ*Aflste20*) showed a significant decrease in growth, sporogenesis, sclerotinogenesis, virulence, and infection compared to the WT (wild type) and complemented strain (Δ*Aflste20^C^*). Further research indicated that Δ*Aflste20* has more sensitivity characteristics than WT and Δ*Aflste20^C^* under various stimuli such as osmotic stress and other types of environmental stresses. Above all, our study showed that the mitogen-activated kinase AflSte20 plays an important role in the growth, conidia production, stress response and sclerotia formation, as well as aflatoxin biosynthesis, in *A. flavus*.

## 1. Introduction

*Aspergillus flavus* is a common saprophytic aerobic fungus. It belongs to the family of Aspergillaceae in the phylum of Ascomycota and is mainly distributed in the warm temperate zone [1]. *A. flavus* is not only a common pathogen of plants but also a human pathogen, and long-term exposure to *A. flavus* or more than 20 other *Aspergillus* species in humans and animals can induce asthma, exogenous alveoli, and allergic bronchial aspergillosis [2,3,4]. During the growth, transportation, and storage of crops, *A. flavus* can induce various crop diseases like ear rot in corn, aflaroot in peanuts, and boll rot in cotton [5]. Food safety issues caused by *A. flavus* are due to its representative secondary metabolites: aflatoxins (AFT), cyclopiazonic acid (CPA), and aflatrem, et al. [1,6,7,8]. Aflatoxin, as a highly toxic fungal toxin with very stable physical and chemical properties, was classified as a first-grade carcinogen by the World Health Organization’s (WHO) international agency for research on cancer in 1993 [9]. Therefore, the signal pathways that affect the regulation of virulence-related factors need to be further explored in *A. flavus*.

Mitogen-activated protein (MAP) kinases are a kind of ubiquitous protein kinases in eukaryotes. The mitogen-activated protein kinase (MAPKs) cascade is regulated by phosphorylation, and its signaling pathway is necessary for cells to adapt to environmental changes [10,11]. In yeast, for example, MAPKs can simultaneously differentiate cellular immune responses and mediate intracellular cascades. Three signal transduction elements, MAPKKK, MAPKK, and MAPK, function sequentially in the kinase cascade [12]. When the cells are stimulated by different environmental factors, the corresponding MAPK cascade is activated. MAPKKK leads to phosphorylation of MAPKK followed by rapid phosphorylation of MAPK by MAPKK [10,13]. In MAPK cascades, the high-osmolarity glycerol (HOG) pathway responding to osmotic stress has caught large attention for a long time. In yeast, the HOG pathway is activated by two different upstream branches, SLN1 and SHO1. Branch SLN1 relies on a two-component system of Sln1, Ypd1, and Ssk1 that activate downstream Ssk2/Ssk22 MAPKKKs. Sho1, Ste20, and Ste11 are required in the Sho1 branch of the HOG pathway to transmit osmotic stress signals. The two branches converge at Pbs2 [13,14,15,16].

In eukaryotes such as fungi, protein kinases play an important role in cell signal transduction [17]. In the budding yeast *Saccharomyces cerevisiae*, Ste20 as a kinase plays a role in the MAPK pathway [18]. It has been found to involve in cell differentiation, growth, mitosis, and apoptosis. In addition, Ste20 participates in MAPK signaling cascades as a response to osmotic stress in *A. flavus* [12]. Ste20 has an N-terminal Cdc42/Rac interactive binding domain (CRIB) and a protein kinase domain on its C-terminal [19]. Previous studies on fungi showed that Ste20 interacts with Cdc42 (small GTPase) and often participates in the cascade reaction of MAPK by phosphorylating Ste11, and regulates many cellular processes such as mycelia development, fungal toxicity, and osmotic stress [19,20,21].

Although the studies of Ste20 has been carried out in some species, the roles of Ste20 have not been clarified until now in *A. flavus*. In this study, we are interested in the role of Ste20 in the regulation of the biological functions of *A. flavus*, including in growth and development, AFB_1_ production, and its response to various environmental stresses. In order to explore the hypothetical role of Ste20 in these processes, the *Aflste20* knockout and complemented mutant strains were constructed. By analyzing the results of experiments, we found that AflSte20 played important roles in asexual development, sclerotia formation, virulence, secondary metabolism, and stress response in *A. flavus*.

## 2. Results

### 2.1. Sequences Analysis of AflSte20 in A. flavus

**Figure 1:**
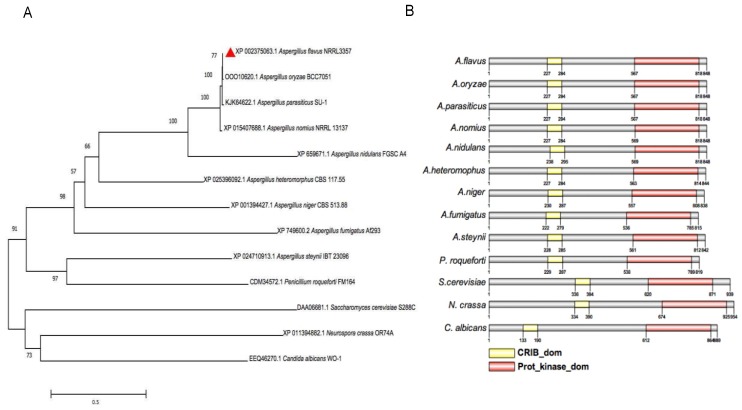
Bioinformatics analysis of the mitogen-activated protein kinase (MAPK) pathway kinase AflSte20 from 13 different fungi: *A. flavus* (XP 002375063.1), *A. oryzae* (OOO10620.1), *A. parasiticus* (KJK64622.1), *A. nomius* (XP_015407688.1), *A. nidulans* (XP_659671.1), *A. heteromorphus* (XP_025396092.1), *A. niger* (XP_001394427.1), *A. fumigatus* (XP_749600.2), *A. steynii* (XP_024710913.1), *Penicillium roqueforti* (CDM34572.1), *S. cerevisiae* (DAA06681.1), *Neurospora crassa* (XP_011394882.1) and *Candida albicans* (EEQ46270.1). (**A**) Phylogenetic relationship of Ste20 homologs from different species was analyzed by MEGA 7.0. Neighbor joining with a bootstrap of 1000 replicates was used to generate the phylogenetic tree. (**B**) Conserved domain analysis of the fungi AflSte20 proteins. InterPro (http://www.ebi.ac.uk/interpro/scan.html) and IBS 1.0 were used in the analysis.

DNA and protein sequences of AflSte20, AFLA_035530, and XP_002375063.1. were obtained from National Center for Biotechnology Information Database (NCBI). AflSte20 was predicted to be a serine/threonine kinase protein with 848 amino acids. The phylogenetic tree was constructed by comparing the sequence of AflSte20 with the amino acid sequences of orthologs of other species, including *A. oryzae*, *A. parasiticus*, *A. nomius*, *A. nidulans*, *A. heteromorphus*, *A. niger*, *A. fumigatus*, *A. steynii*, *Penicillium roqueforti*, *S. cerevisiae*, *Neurospora crassa* and *Candida albicans*, which were retrieved from NCBI (http://www.ncbi.nlm.nih.gov). The evolutionary relationship of these proteins was analyzed by the software Molecular Evolutionary Genetic Analysis Version 7.0.26 (MEGA 7) (Figure 1A). Domains in Ste20 were further analyzed by using the software SMART and IBS 1.0 (Figure 1B). After domain analysis of AflSte20 protein, it was found that there were two domains, including CRIB_dom (Cdc42/Rac interactive binding motif domain) and Prot_kinase_dom (Protein Kinase domain). Compared to other homologous proteins, the CRIB_dom and Prot_kinase_dom were found to be highly conserved in the fungal species analyzed.

### 2.2. AflSte20 is Involved in Vegetative Growth and Conidial Production

In order to explore the influence of *Aflste20* on *A. flavus*, the knockout mutant strain (Δ*Aflste20*) was constructed by a homologous recombination principle (Figure S1A). Whereas, in order to certify the role of *Aflste20* and to confirm that the phenotypic effects observed were exclusively due to the deletion of *Aflste20*, we created the *Aflste20* complementation strain. PCR verification of the obtained strains showed that ORF (Open Reading Frame) could be detected in WT (wild type) and ∆*Aflste20^C^*, but not in Δ*Aflste20*, while AP (the fusion PCR product by 5’UTR of the *Aflste20* gene and part of *pyrG*) and BP (the fusion PCR product by 3’UTR of the *Aflste20* gene and part of *pyrG*) bands with the correct size could be detected in Δ*Aflste20* and ∆*Aflste20^C^* (Figure S1B), proving a successful generation of the knockout strain. RT-PCR and qRT-PCR tests further verified the expression of the Aflste20 transcript in the WT and complementation strain but not in the null mutant, confirming that the targeted gene had been deleted. (Figure S1C,D).

**Figure 2:**
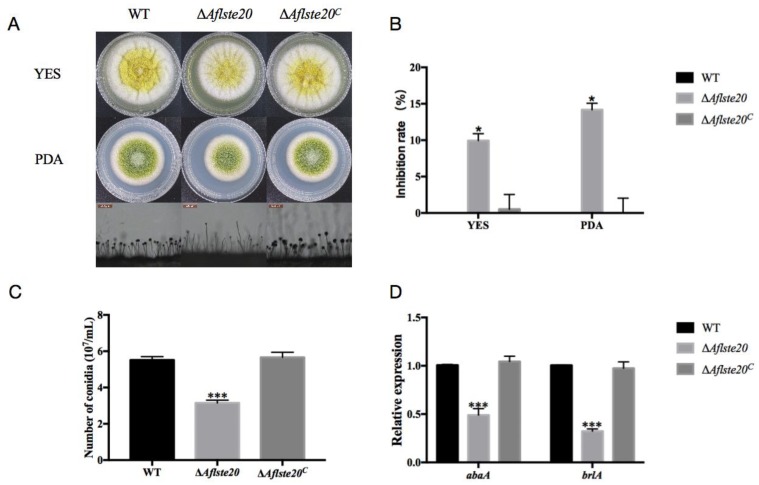
The function of AflSte20 in vegetative growth and conidial production in *A. flavus*. (**A**) The phenotype of wild type (WT), Δ*Aflste20,* and Δ*Aflste20^C^* strains. These strains were grown on YES/PDA medium at 37 °C for 72 h and the conidiophores were observed under microscope (200×). (**B**) The growth inhibition rate of strains was calculated based on the result of (A), according to the formula: growth inhibition rate = [(diameter of WT strains colony—diameter of Δ*Aflste20* or Δ*Aflste20^C^* strains colony/diameter of WT strains colony)] × 100. (**C**) The conidia number of WT, Δ*Aflste20,* and Δ*Aflste20^C^* strains were calculated by hemocytometer. (**D**) The expression level of *abaA* and *brlA* genes in *A. flavus* by qRT-PCR analysis. For panels (B), (C), and (D), each bar indicates the mean ± standard deviation (SD) of five replicate assay experiments. * represents significant difference (*p* < 0.05), *** represents significant difference (*p* < 0.001).

To verify the function of the *Aflste20* gene in the growth process of *A. flavus*, WT, Δ*Aflste20*, and Δ*Aflste20^C^* strains were inoculated on potato dextrose agar (PDA) and Yeast Extract Sucrose (YES) media, then grown at 37 °C for 4 days in the dark. The results showed that under the same culture conditions, the colony diameter of the Δ*Aflste20* mutant was restrained dramatically (*p* < 0.05; *n* = 5) compared to Δ*Aflste20* and WT strains. The growth inhibition rate of Δ*Aflste20* were 9.94% and 14.18% in YES and PDA media respectively. Under the microscope, the conidiophores of Δ*Aflste20* were observed to be more sparsely distributed than that of WT and Δ*Aflste20^C^* (Figure 2A,B). Also, the statistical results indicated that compared to WT (5.52 × 10^7^/mL) and Δ*Aflste20^C^* (5.66 × 10^7^/mL) strains, the amount of conidia produced by the Δ*Aflste20* mutant (3.15 × 10^7^/mL) were significantly decreased on PDA (*p* < 0.001; *n* = 5) (Figure 2C). qRT-PCR analysis of *brlA* (a regulatory gene in primary regulation of asexual development) and *abaA* (an essential gene for both differentiation and function of phialides, and is activated by BrlA during the middle stages of conidiophores development [22,23]) genes showed that the relative expression of the *abaA* and *brlA* gene in Δ*Aflste20* (*abaA*: 48.9%; *brlA*: 32.3%) was significantly (*p* < 0.001; *n* = 5) lower than that of WT and Δ*Aflste20^C^* (Figure 2D). All the above results suggest that AflSte20 plays a positive regulatory role in the control of growth and asexual sporulation of *A. flavus*.

### 2.3. AflSte20 Mediates Sclerotia Formation in A. flavus

**Figure 3:**
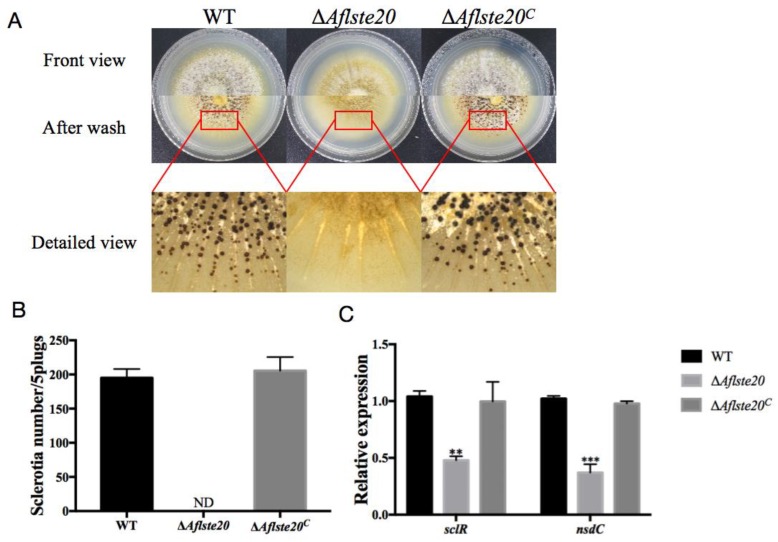
The role of *Aflste20* in sclerotia formation in *A. flavus*. (**A**) Phenotypes of the WT, ∆*Aflste20,* and ∆*Aflste20^C^* strains were observed after grown on a WKM medium for 7 days in the dark. The mycelia were cleaned with 75% ethanol to make sclerotia visible. (**B**) The number of sclerotia from WT, Δ*Aflste20,* and Δ*Aflste20^C^* strains. (**C**) The expression level of *sclR* and *nsdC* genes in *A. flavus* by qRT-PCR analysis. For panels (B) and (C), each bar indicates the mean ± standard deviation (SD) of three replicate assay experiments. ** represents significant difference (*p* < 0.01), and *** represents significant difference (*p* < 0.001).

*A. flavus* relies on the production of sclerotia, which are considered to be the resistant structures that act as a repository for sexual spore and allow the fungi to survive in the adverse living environment [24]. To explore the influence of the *Aflste20* gene on the production of sclerotia by *A. flavus*, 10^4^ asexual spores were inoculated on the Wickerham (WKM) medium and cultured in the dark for seven days. The number of sclerotia was counted after cleaning mycelia with 75% alcohol (Figure 3A). The results showed that the sclerotia production by Δ*Aflste20* (0 per 5 plugs) was significantly impaired (*p* < 0.001; *n* = 5) compared to that of WT (195.2 per 5 plugs) and Δ*Aflste20^C^* (205.6 per 5 plugs) strains (Figure 3B). qRT-PCR was carried out to check the expression level of *sclR* and *nsdC* genes, which are the positive regulators of sclerotia formation [25]. And the results showed a downward trend in the knockout mutant Δ*Aflste20* strain of *sclR* (46.08%, *p* < 0.01; *n* = 5) and *nsdC* (36.28%, *p* < 0.001; *n* = 5) (Figure 3C). All of these results suggest that AflSte20 positively regulates the sclerotia production in *A. flavus*.

### 2.4. AflSte20 Participates in the Response to Different Stresses in A. flavus

**Figure 4:**
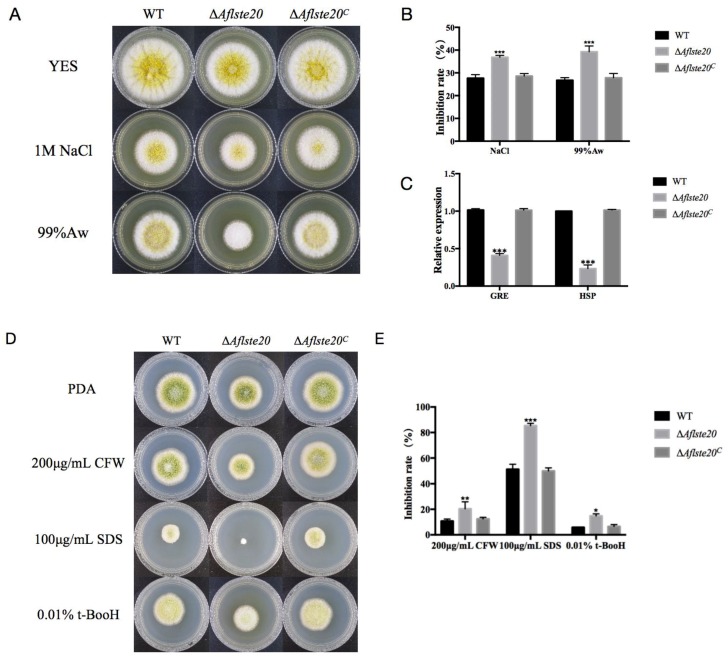
Phenotype and inhibition rate of strains under different stress conditions. (**A**) Morphology of WT, ∆*Aflste20,* and ∆*Aflste20^C^* strains under hyperosmotic stress. (**B**) The inhibition of osmotic stress on the mycelium growth of each strain. (**C**) qRT-PCR analysis of expression level of the genes (*HSP* and *GRE*) related with osmolality. (**D**) The colony of each strain under cell wall stress and oxidation stress. (**E**) The mycelial growth inhibition of all strains under cell wall stress and oxidation stress. For panels (B), (C), and (E), each bar indicates the mean ± standard deviation (SD) of five replicate assay experiments. * refers to significant difference (*p* < 0.05), ** represents significant difference (*p* < 0.01), and *** represents significant difference (*p* < 0.001).

Previous studies elaborated that the *ste20* gene is involved in MAPK pathway in yeast [12]. Due to a predicted role of AflSte20 in signal transduction previously [26], we analyzed whether *Aflste20* takes part in the response to a variety of environmental stress conditions such as hyperosmotic pressure, oxidation stress, and cell wall damage. In this study, first we analyzed the effect of AflSte20 under osmotic stress. We found that, in the presence of osmotic stress induced by 1M NaCl or 99% water activity, the growth was significantly inhibited compared to the WT and complementation strains (*p* < 0.001; *n* = 5), indicated that Δ*Aflste20* mutant was more sensitive to osmotic stress (Figure 4A,B). Subsequently, the expression level of HSP and GRE genes which are related with osmolality were determined, the result indicated that the expression levels of the *HSP* and *GRE* genes were significantly (*p* < 0.001; *n* = 5) decreased to 59.05% and 76.70%, respectively, in Δ*Aflste20* (Figure 4C). Similarly, the Δ*Aflste20* mutant also showed a higher inhibition rate which was about 253% of the wild type under oxidative stress with 0.01% t-BooH [(CAS number: 75-91-2) (*p* < 0.01; *n* = 5)], 166% and 189% of the wild type under cell wall integrity damage with 100 μg/mL lauryl sodium sulfate (SDS) (*p* < 0.001; *n* = 5) and with 200 μg/mL calcofuor white (CFW) (*p* < 0.05; *n* = 5), respectively (Figure 4D,E). Overall, these results suggest that Δ*Aflste20* does not only participate in the response of *A. flavus* to hyperosmotic stress, but also to oxidation stress and cell wall stress.

### 2.5. AflSte20 Positively Regulates Aflatoxin Biosynthesis

**Figure 5:**
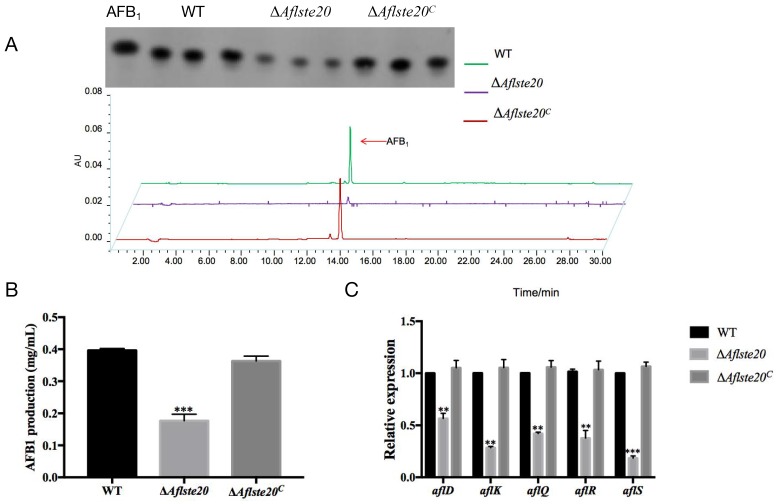
Role of *Aflste20* in aflatoxin biosynthesis. (**A**) thin-layer chromatography (TLC) and high-performance liquid chromatography (HPLC) analysis of AFB_1_ production in WT, ∆*Aflste20,* and ∆*Aflste20^C^* strains after 7 days of incubation. (**B**) Relative AFB_1_ production was calculated from (A). (**C**) The qRT-PCR analysis of the expression of key structure genes (*aflD*, *aflK*, *aflQ*, *aflR,* and *aflS*) in *A. flavus*. Bars in panels (B) and (C) indicate the mean ± standard deviation (SD) of assays performed three times. ** represents significant difference (*p* < 0.01), and *** represents significant difference (*p* < 0.001).

In order to evaluate whether AflSte20 plays a function in the regulation of aflatoxin biosynthesis. AFB_1_ was extracted from WT, Δ*Aflste20,* and Δ*Aflste20^C^* strains in the YES liquid medium after incubation for 6 days at 29 °C and detected by TLC (Thin Layer Chromatograph) (Figure 5A). According to the TLC result, the AFB_1_ production of Δ*Aflste20* strains was decreased more than one half when compared with WT strains (*p* < 0.001; *n* = 5) (Figure 5B). Simultaneously, the expression of regulatory genes, *aflR* and *aflS*, and key structural genes including *aflD*, *aflK,* and *aflQ* in the aflatoxin biosynthesis cluster, were severely repressed in the Δ*Aflste20* mutant strain (*aflD*: 56.53%; *aflK*: 28.42%; *aflQ*: 42.10%; *aflR*: 37.63%; *aflS*: 18.38%) (Figure 5C). All the above results confirmed that AflSte20 could positively regulate AFB_1_ production in *A. flavus*.

### 2.6. AflSte20 Has an Influence on Conidiation and Aflatoxin Biosynthesis to Crop Seeds

**Figure 6:**
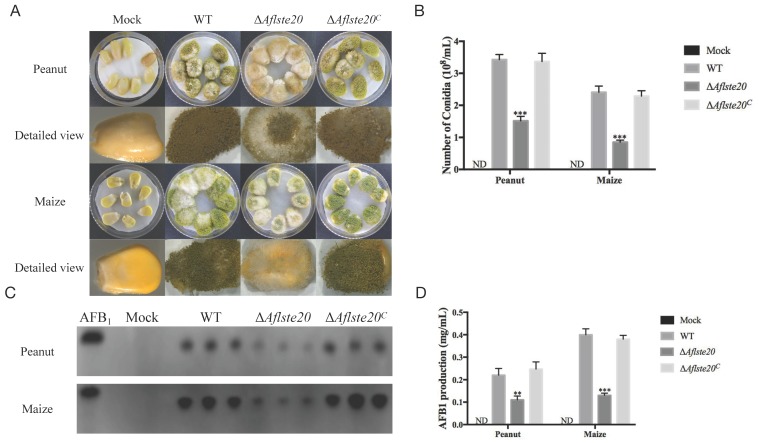
The effect of *Aflste20* deletion on conidiation and aflatoxin biosynthesis to crops. (**A**) The peanut and maize kernels were inoculated with WT, ∆*Aflste20,* and ∆*Aflste20^C^* strains. (**B**) Conidia production of infected peanut and maize seeds. (**C**) Aflatoxin was analyzed by TLC, which was extracted from infected peanut and maize kernel seeds. (**D**) The relative AFB_1_ production was analyzed from (C). For panels B and D, each bar indicates the mean ± standard deviation (SD) of assays performed three times. ** represents significant difference (*p* < 0.01), and *** represents significant difference (*p* < 0.001).

As a common pathogenic fungus, *A. flavus* is particularly harmful to crops. To explore the effect of AflSte20 in *A. flavus* on the conidial production and aflatoxin biosynthesis in the infection process, the seeds of crops were infected with Δ*Aflste20*, WT, and Δ*Aflste20^C^* strains and grown in dark for seven days (Figure 6A). The ability of different strains to produce asexual spores and AFB_1_ during infection was determined (Figure 6A,C). According to the experimental results, we found that the spore production of Δ*Aflste20* strains infection were significantly decreased to about 44.31% (peanut) and 35.27% (maize) when compared to WT (*p* < 0.001; *n* = 5). We also found the AFB_1_ production of Δ*Aflste20* strains after infection was significantly reduced to about 50.00% (peanut, *p* < 0.01; *n* = 5) and 32.50% (maize, *p* < 0.001; *n* = 5) of the wild type (Figure 6B–D). The results demonstrated that the ability of *A. flavus* to infect crops decreased significantly when the *Aflste20* gene was absent. All of these results indicate that the *Aflste20* gene was important for *A. flavus* to maintain its ability for asexual spore production and aflatoxin biosynthesis when infecting crop seeds such as peanuts and maize.

## 3. Discussion

Previous studies found that the MAPK pathway plays an important role in eukaryotes, and that the cascade of MAPK is involved in apoptosis, growth regulation, hyperosmosis regulation, cell tolerance, gene expression, cell division, and ascospore development [11]. As a member of the protein kinase family, the yeast p21-activated protein kinase(PAK) homolog Ste20 is essential for the Sho1-dependent activation of the Hog1 MAP kinase in response to severe osmotic stress, and this function of Ste20 in the HOG pathway requires binding of the small GTPase Cdc42 [27,28], but until now, no study on Ste20 homologues in *A. flavus* had been reported. In this study, we found that the *Aflste20* gene consists of 2544 nucleotide residues. The comparison of amino acid sequences with other selected orthologues revealed that AflSte20 in *A. flavus* showed the lowest identity (46.18%) to *S. cerevisiae*, and had the highest identity (99.76%) to *A. oryzae* (Figure 1). A protein kinase domain in the C-terminal of AflSte20 was highly conserved between AflSte20 and its orthologues. In recent studies, Ste20 orthologues have been increasingly reported to play important roles in different fungi. In yeast, the activated Ste20 can bind with the SH3 domain of Sho1 to promote the activation of Hog1 [29], and it also participates in the mycelium growth, colony reproduction, and other life activities [27]. In this study, we investigated the effects of AflSte20 mutations on the fungal biology and aflatoxin biosynthesis of *A. flavus.* Firstly, we found that the Δ*Aflste20* strain was significantly inhibited in vegetative growth, and conidiation occurred independently of the culture used or the surface of crops. Our results suggested that AflSte20 might play an important regulatory role in the growth and asexual reproduction in *A. flavus*. On the other hand, some fungi produce sclerotium, a sexual reproductive and survival structure, to adapt to adverse environmental conditions [30]. Besides impacting the function of growth and development, the *Aflste20* deletion mutant also exhibited observable effects on sclerotia production. Compared to WT and Δ*Aflste20^C^*, our results showed that sclerotia production was almost blocked in the *Aflste20* deletion mutant. Simultaneously, we also found that the expression of the sexual development-related genes, *sclR* and *nsdC*, were prominently suppressed in the Δ*Aflste20*. Thus, for the first time, the result demonstrates that AflSte20 positively regulates sexual reproduction and would be a key factor to make fungi survive under stress conditions in *A. flavus.* Together, these results suggested that AflSte20 could control, directly or indirectly, both asexual and sexual developmental cycles. Compared to the media used, we found that if there were enough carbon sources in the environment, AflSte20 involved in the asexual reproduction and activated BrlA and AbaA. On the contrary, with carbon starvation, AflSte20 also works but activated sexual development which induced the production of sclerotia. Thus, the role of AflSte20 in the asexual and sexual developmental cycles might depend on the outside ambient and overlap in the carbon starvation pathway. To resolve this confusion, further research is urgently needed.

MAPK cascades have been found in different fungi, such as *Fusarium spp.*, *Aspergillus spp.*, *M. oryzae*, *B. cinerea*, and so on. In *A. nidulans*, two MAPK pathways (Hog1-MAPK and Slt2-MAPK) have been found to be involved in many external environmental stresses such as osmotic pressure and cell wall stress [31,32,33,34,35]. In the present study, we found that AflSte20 could play vital functions in responses to hyperosmotic stress and positively correlated with mycelia development. Our findings were reliable based on the previous study on yeast which showed that *ste20* gene in the HOG pathway plays an essential role in response to osmotic stress [12]. Studies on *Candida glabrata* revealed that Ste20 is necessary in response to osmotic stress and maintains a complete cell wall integrity pathway [36]. Thus, we were also interested in whether AflSte20 would respond to cell wall stress in *A. flavus*. As expected, consistent with the effect of Ste20 in *C. glabrata*, the loss of *Aflste20* also leads to the increased sensitivity to cell wall stress in *A. flavus*. Furthermore, in *S. cerevisiae*, when the protein on HOG pathway is absent, it is found that the mutant strain is more sensitive to oxidative stress [37]. We also wondered whether AflSte20 has a crosstalk with other pathways and plays a role in responding to oxidative stress. In fact, as AflSte20 performed various metabolic processes, the highly reduced tolerance of the ∆*Aflste20* strain supports that AflSte20 might play an important role in the response to oxidative stress and functioning when oxidative damage occurs. One possible explanation for this phenomenon is that AflSte20, which works in the HOG pathway, has a crosstalk with an intact cell wall integrity pathway or other signaling pathways related to oxidative stress. An alternative possibility is that AflSte20, with a regulatory CRIB domain on the N terminal, facilitates the kinase to interact directly with all kinds of signaling molecules and regulatory proteins [38].

In the pathogenic fungus *C. albicans*, Cla4 is a protein in the Ste20p family, and this protein deficiency results in reduced toxicity and also inhibits the toxicity of *C. albicans* in the mouse model [39]. It is also worth mentioning that although the Ste20 has caught great attention for a long time, the comprehension about its pathogenic function in *A. flavus* is still a puzzle. So, we focused on the aflatoxin production and infection ability of AflSte20 to crops. AFB_1_, as a secondary metabolite of *A. flavus*, is a key factor correlated with the resistance to reactive oxygen species (ROS) during infection and enhancing its virulence on plants [40,41]. A significant decrease of AFB_1_ with reduced infection ability of ∆*Aflste20* to crops suggests that at least one of the reasons affecting the virulence of *A. flavus* is due to the *Aflste20* gene which can regulate the key genes in the aflatoxin biosynthesis-relevant cluster (Figure 6C).

In summary, our experiments demonstrated that AflSte20 is involved in many physiological activities such as the growth and development of *A. flavus*, the production of secondary metabolites and crop contamination of *A. flavus*, and plays an important role in resisting many external stresses such as hyperosmosis stress. Above all, this work may provide ideas and references for the prevention and control of *A. flavus* pollution.

## 4. Materials and Methods

### 4.1. Strains and Culture Conditions

All strains used in this study were given in Table 1. All strains were cultivated on potato dextrose agar (PDA, BD Difco, USA), YES medium and WKM agar medium at 37 °C or 29 °C for growth assay and sclerotia assay. Each strain was cultivated on five plates, and all experiments were repeated three times.

### 4.2. Bioinformatics Analysis

Gene sequence (Accession number AFLA_035530) and protein sequence (ID XP_002375063.1) of *Aflste20* were downloaded from NCBI (http://www.ncbi.nlm.nih.gov). The phylogenetic tree was created by MEGA 7 with different protein sequences of other Ste20 orthologs downloaded from NCBI. The domains of these proteins were analyzed with InterPro (http://www.ebi.ac.uk/interpro/scan.html) and edited with IBS 1.0.

### 4.3. Gene Deletion and Complementation

The homologous recombination method was used to obtain the deletion mutant Δ*Aflste20* [42]. Three fragments (1194 bp 5’-UTR of *Aflste20*, 1038 bp 3’-UTR of *Aflste20* and 1890 bp *pyrG* gene) were amplified and then connected by overlap PCR. The fragments were then transferred into the protoplasts of *A. flavus* CA14. For the complemented strains [42], firstly, the *ste20* fragment in *A. flavus* was amplified with *ste20*-C-p1/*ste20*-C-p4 primers, then transferred into the ∆*Aflste20* protoplasts with 2 mg/mL 5-FOA (5-fluoroorotic acid) to substitute *pyrG* in Δ*Aflste20*. Lastly, the *pyrG* gene was inserted behind the *ste20* to get the *pyrG* prototroph complemented strains (∆*Aflste20^C^*) via a homologous recombination. The complemented strain was confirmed by PCR. With an *actin* gene as the inner reference, both of the verified ∆*Aflste20* mutant and ∆*Aflste20^C^* strains were also verified by qRT-PCR assay. The primers used in the study are listed in Table 2 and Table 3.

### 4.4. Measurement of Conidia, Conidiophores, and Sclerotia

In order to determine the quality and amount of conidia, conidiophores, and sclerotia, 10^4^ conidia were added to YES, PDA, and WKM solid media, respectively (10 mL media for one plate), and grown at 37 °C in the dark. The colony diameter was measured after five days. Conidia were dispersed in 3mL of 0.05% Tween-20 water solution, then the number of conidia were calculated by using a hemocytometer. For conidiophores investigation, the hyphae were scraped and cut as a rectangle with solid media by a surgical blade. Then these media with hyphae were added onto a glass slide and cultivated at 37 °C overnight. Conidiophores were observed through a light microscope (Magnification scale, 200×). For sclerotia formations analysis, WKM solid medium was used to induce the production of sclerotia. WT, Δ*Aflste20,* and Δ*Aflste20^C^* strains (10^7^ spores/mL) were incubated onto WKM media (1 μL for one plate) at 37 °C in the dark for seven days, and sclerotia were counted by the method described in detail by Yang et al. [43].

### 4.5. Stress Assay

To understand the role of *Aflste20* gene response to multiple stress in *A. flavus*, the WT, Δ*Aflste20,* and Δ*Aflste20^C^* strains were cultured onto PDA agar medium at 37 °C in the dark for 4 days with an oxidative stress agent (0.01% t-BooH), hyperosmotic stress agent (1 M NaCl and 99% water activity), cell membrane stress agent (100 μg/mL, SDS), and cell wall stress agent (200 μg/mL CFW) obtained from Sigma-Aldrich, St. Louis., MO, USA. To determine the effect of Ste20 on the stress response of *A. flavus*, the relative growth inhibition rates were analyzed using the formula as followed: growth inhibition rate = [(diameter of the colony without inhibitor—diameter of colony with inhibitor)/diameter of colony without inhibitor] x100. The assay was repeated at least three times.

### 4.6. Determination of AFB1 Production

For the thin-layer chromatography (TLC) analysis and high-performance liquid chromatography (HPLC) analysis of aflatoxins (AF) production, WT, Δ*Aflste20,* and Δ*Aflste20^C^* strains (10^7^ spores/mL) were inoculated into 10 mL YES liquid media at 29 °C in dark for seven days, (30μL spore suspension per plate). After that, aflatoxin was extracted using a method previously described by Yang et al. [44]. The supernatant samples were analyzed by TLC with a solvent system (chloroform: acetone = 9:1) and detected by ultraviolet light. The quantity of the AF production was analyzed by the Gene Tools software. For HPLC analysis, extracted AF was detected by an HPLC system (Waters, Milford, MA, U.S.A.) on a MYCOTOX reversed-phase C18 column (4.6 × 250 mm, Pickering Laboratories, USA) according to a method previously described [45].

### 4.7. Conidiation and Aflatoxin Biosynthesis of A. flavus in Crop Seeds

The method to detect asexual spore production and aflatoxin biosynthesis of *A. flavus* in crop seeds was performed according to the previously illustrated procedure by Hu et al. [42]. Briefly, the crop kernels were washed with 0.05% sodium hypochlorite (Shanghai Bojing Chemical Co.,Ltd., Shanghai, China) and the peanut and corn seeds were put into a suspension with 10^5^ spore/mL conidia of each strain in a rotary shaker at 80 r/min for 30 min. Viable cotyledon was dried and laid on a sterile petri dish plate to cultivate at 29 °C in dark for 7 days.

### 4.8. Quantitative Real-Time Polymerase Chain Reaction (qRT-PCR)

To determine the expression level of the genes related to different phenotypes. *A. flavus* WT, Δ*Aflste20,* and Δ*Aflste20^C^* strains were inoculated on YES medium and grown for 72 h at 37 °C in the dark, then mycelium was collected. Total RNA was extracted from 0.1 g mycelium using an RNA isolation kit (Promega, Madison, WI, USA). And cDNA was synthesized using a First Strand cDNA Synthesis Kit (TransGen Biotech, Beijing, China). Then, as a template, cDNA was used for qRT-PCR amplification with SYBR Green qRT-PCR mix (TaKaRa Biotechnology, Japan) in a PikoReal Real-Time PCR system (Thermo Fisher Scientific, USA). The expression levels of target genes were calculated by the 2^-ΔΔCt^ method [46]. All the primers used in this experiment were listed in Table 3. All the experiments were performed three times.

### 4.9. Statistical Analysis

The data for all the experiments were expressed as means ± standard deviation. At least three biological replicates were used for analysis. Tukey’s multiple comparison test was used to compare the difference between each treatment and the control. Significance statistical analysis was performed using the GraphPad Prism 5 and recognized as significant when *p*-values were < 0.05.

## Figures and Tables

**Table 1:** Strains used in the study.

Strains	Genotype Description	Source
*A. flavus* CA14	∆*ku70*, ∆*pyrG*	Purchased from FGSC
wild-type (WT)	∆*ku70*, ∆*pyrG*::*AfpyrG*	This study
∆*Aflste20*	∆*ku70*, ∆*pyrG*::*AfpyrG*, ∆*Aflste20*	This study
∆*Aflste20^C^*	∆*ku70*, ∆*Aflste20*::*Aflste20*, ∆*pyrG*::*AfpyrG*	This study

**Table 2:** Primers used for the construction of strains in this study.

Primer Name	Sequence (5’–3’)	Amplified Fragment
*ste20-p1*	TGCTTAGAGGATGGGATT	5’UTR of *Aflste20*
*ste20-p3*	GGGTGAAGAGCATTGTTTGAGGCCGCTAGGCTCAGTGATGG	
*ste20-p6*	GCATCAGTGCCTCCTCTCAGACCCAGATACCCATTGCTCC	3’UTR of *Aflste20*
*ste20-p8*	CACCAGCCCACATAGAAT	
*ste20-p4*	GCCTCAAACAATGCTCTTCACCC	*A. fumigatus pyrG*
*ste20-p5*	GTCTGAGAGGAGGCACTGATGC	
*ste20-p2*	TTTGGCACTCGCTTGTCC	Fusion PCR
*ste20-p7*	CCGCTCAAGTCTGGGTTA	
*ste20-p9*	TCGGTTAATCACATCTGTCTC	ORF validates primers
*ste20-p10*	CATCAATCATCGCCATCTA	
*pyrG-801*	CAGGAGTTCTCGGGTTGTCG	AP BP
*pyrG-1020*	CAGAGTATGCGGCAAGTCA	
*ste20-C-p1*	GCTTCTGGTGGCGTATT	5’UTR of ∆*Aflste20^C^*
*ste20-C-p2*	GGGTGAAGAGCATTGTTTGAGGCCGCTCAAGTCTGGGTTAT	
*ste20-C-p3*	GCATCAGTGCCTCCTCTCAGACCTGATGTCTCGCCTGTTA	3’UTR of ∆*Aflste20^C^*
*ste20-C-p4*	CTCCGCCGCAACTTTAT	
*ste20-C-p5*	CTCTGGGTGGTGATGGA	Fusion PCR
*ste20-C-p6*	AGAATACGAGGCTTGTGG	

**Table 3:** Primers used for amplifying the related genes in this study.

Gene	Forward Sequences (5’–3’)	Reverse Sequences (5’–3’)
*Aflste20*	ATCAACGACTCGCACAATAA	GCTCGCCCTCAATCATCT
*brlA*	GCCTCCAGCGTCAACCTTC	TCTCTTCAAATGCTCTTGCCTC
*abaA*	TCTTCGGTTGATGGATGATTTC	CCGTTGGGAGGCTGGGT
*nsdC*	GCCAGACTTGCCAATCAC	CATCCACCTTGCCCTTTA
*sclR*	CAATGAGCCTATGGGAGTGG	ATCTTCGCCCGAGTGGTT
*aflR*	AAAGCACCCTGTCTTCCCTAAC	GAAGAGGTGGGTCAGTGTTTGTAG
*aflS*	CGAGTCGCTCAGGCGCTCAA	GCTCAGACTGACCGCCGCTC
*aflD*	GTGGTGGTTGCCAATGCG	CTGAAACAGTAGGACGGGAGC
*aflK*	GAGCGACAGGAGTAACCGTAAG	CCGATTCCAGACACCATTAGCA
*aflQ*	GTCGCATATGCCCCGGTCGG	GGCAACCAGTCGGGTTCCGG
*actin*	ACGGTGTCGTCACAAACTGG	CGGTTGGACTTAGGGTTGATAG
*GRE*	GCGTATCGTCGTTACCTCATC	CCTTCTCCTTTACCTCCTCGAT
*HSP*	CCGGCATACTATGTCTCGTCT	TAGGGCCTTCGTCGAACA

## Supplementary Materials

The following are available online at https://www.mdpi.com/2072-6651/11/12/730/s1, Figure S1: Construction strategy and confirmation of the *Aflste20* knockout and complemented strains.
