# Supplementary material for: AflSte20 Regulates Morphogenesis, Stress Response, and Aflatoxin Biosynthesis of Aspergillus flavus
Source: Toxins (Basel). 2019 Dec 13;11(12):730. doi: 10.3390/toxins11120730 (PMC6950481; doi:10.3390/toxins11120730)
Supplement: Supplementary file 1 [file toxins-11-00730-s001.pdf]

Supplementary Materials: AflSte20 Regulates Morphogenesis, Stress Response and Aflatoxin Biosynthesis of *Aspergillus flavus*

Ding Li, Ling Qin, Yinchun Wang, Qingchen Xie, Na Li, Shihua Wang and Jun Yuan

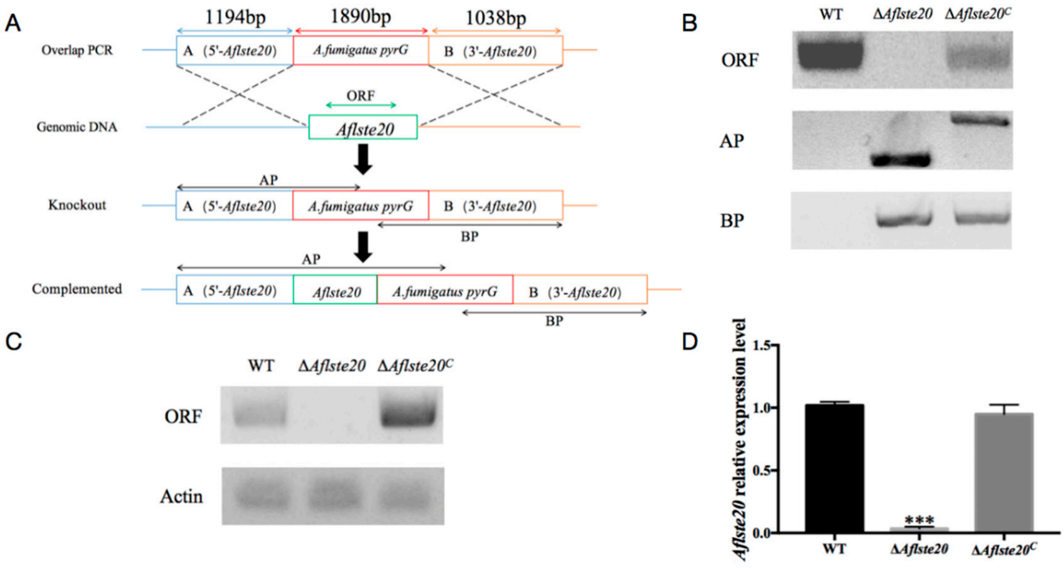

Figure S1. Construction strategy and confirmation of the *Aflste20* knock-out and complemented strains. (**A**) The scheme of *Aflste20* deletion strategy. (**B**) Confirmation of *Aflste20* mutants by PCR analysis with gDNA as template. (**C**) Verification of WT, Δ*Aflste20* and Δ*Aflste20*^{C} strains by RT-PCR analysis with cDNA as template. (**D**) The expression level of *Aflste20* in WT, Δ*Aflste20* and Δ*Aflste20*^{C} strains by qRT-PCR. *** represents significant difference ($p < 0.001$).
